# Ecological consequences of antimicrobial residues and bioactive chemicals on antimicrobial resistance in agroecosystems

**DOI:** 10.1016/j.jare.2024.10.013

**Published:** 2024-10-15

**Authors:** Muhammad Shafiq, Charles Obinwanne Okoye, Mudasir Nazar, Wajid Ali Khattak, Abdelazeem M. Algammal

**Affiliations:** a Research Institute of Clinical Pharmacy, Department of Pharmacology, Shantou University Medical College, Shantou 515041, China; b Biofuels Institute, School of Environment & Safety Engineering, Jiangsu University, Zhenjiang 212013, China; c School of Life Sciences, Jiangsu University, Zhenjiang 212013, China; d Department of Zoology & Environmental Biology, University of Nigeria, Nsukka 410001, Nigeria; e Institute of Animal Science, Jiangsu Academy of Agricultural Science, Nanjing 210014, China; f College of Life Sciences and Oceanography, Shenzhen University, Shenzhen 518060, China; g Department of Bacteriology, Immunology, and Mycology, Faculty of Veterinary Medicine, Suez Canal University, Ismailia 41522, Egypt

**Keywords:** Agroecosystems, Antimicrobial resistance, Antimicrobial residues, Bioactive chemicals, Ecological risks

## Abstract

**Background:**

The widespread use of antimicrobials in agriculture, coupled with bioactive chemicals like pesticides and growth-promoting agents, has accelerated the global crisis of antimicrobial resistance (AMR). Agroecosystems provides a platform in the evolution and dissemination of antibiotic-resistant bacteria (ARB) and antibiotic resistance genes (ARGs), which pose significant threats to both environmental and public health.

**Aim of review:**

This review explores the ecological consequences of antimicrobial residues and bioactive chemicals in agroecosystems, with a focus on their role in shaping AMR. It delves into the mechanisms by which these substances enter agricultural environments, their interactions with soil microbiomes, and the subsequent impacts on microbial community structure.

**Key scientific concepts of review:**

Evidence indicates that the accumulation of antimicrobials promotes resistance gene transfer among microorganisms, potentially compromising ecosystem health and agricultural productivity. By synthesizing current research, we identify critical gaps in knowledge and propose strategies for mitigating the ecological risks associated with antimicrobial residues. Moreover, this review highlights the urgent need for integrated management approaches to preserve ecosystem health and combat the spread of AMR in agricultural settings.

## Highlights

• Antibiotic resistance in agroecosystems emanates from environmental antibiotic residues.
• Agroecosystems contain persistent levels of antibiotics and bioactive chemicals.
• Interactive impacts of antibiotics and bioactive chemicals on agricultural ecosystems are largely unknown.
• Antibiotic-resistant bacteria and genes pose an emerging risk to ecological health.
• Field studies are crucial for understanding the spread and removal of antibiotic residues in agroecosystems.

## Graphical abstract

**Figure:**
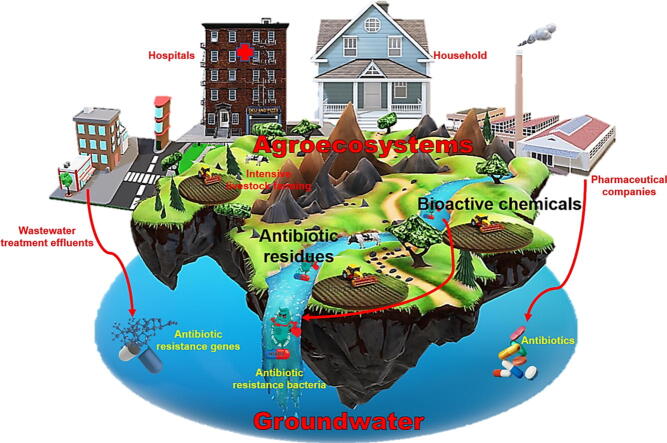

## Introduction

Antimicrobials include antibiotics, antivirals, antifungals, and antiparasitics frequently used in veterinary and human medicine to prevent and treat infections, manage farm animals, and crop protection [1]. The antibiotic era was signaled when penicillin was discovered in 1928, which helped save countless lives during World War II [2]. Antibiotic efficacy has been diminished, and the advent of multidrug-resistant bacteria seriously threatens human health because of the unchecked and careless use of antibiotics [3], [4], [5]. Researchers agree that antibiotic misuse or overuse is the primary factor contributing to the establishment of antimicrobial resistance. Antimicrobial resistance (AMR) is a global health crisis with far-reaching implications for human and environmental well-being [6]. While much attention has been directed toward the clinical domain, the agroecosystem has emerged as a crucial arena for understanding the intricate dynamics of antimicrobial resistance and its potential consequences [7].

The extensive use of antimicrobials in agriculture for disease prevention and growth promotion has been a common practice for decades, but this overreliance fueled the evolution and spread of antimicrobial resistance [8]. Agroecosystems are dynamic environments where various components like, soil, water, and crops interact with antibiotic residues and bioactive chemicals. Globally, soils, sediments, and agricultural products are increasingly found to contain contaminants of emerging concern (CECs), such as hormones, pesticides, antimicrobials, antibiotic-resistant bacteria (ARB), and antibiotic-resistant genes (ARGs) [9], [10], [11]. This prevalence is largely due to the widespread use of pharmaceuticals in both animal husbandry and human healthcare, coupled with environmental contamination from wastewater discharge, application of animal manure, and sewage sludge. Recent years have seen growing scientific interest in the complex interactions between bioactive chemicals and antimicrobials in agricultural settings, including how these factors shape antimicrobial resistance (AMR) and contribute to the selection and persistence of ARB and ARGs [12]. These interactions create a reservoir that poses potential risks to human health through various pathways [13].

Antimicrobials often coexist with bioactive chemicals such as phytochemicals, hormones, enzymes, pheromones, steroids, peptides, biopesticides, pesticides, plant growth promoters, allelopathic compounds, essential oils, and biostimulants regularly used in agroecosystems [14]. The use of these bioactive chemicals in agroecosystems aligns with the principles of sustainable and environmentally friendly agriculture, which could play a pivotal role in shaping the microbial landscape of the agroecosystem [15]. Moreover, introducing these chemicals can disrupt the intricate balance between beneficial and pathogenic microorganisms, creating selective pressures that drive the evolution of antimicrobial resistance [16]. Some recent studies have shown that exposure to sub-lethal concentrations of pesticides can enhance the transfer of ARGs among bacteria, facilitating the development of multidrug resistance [17], [18], [19]. This phenomenon underscores the urgent need to understand antimicrobials, bioactive chemicals, other emerging contaminants, and heavy metals interact and influence antimicrobial resistance dynamics within the agroecosystem.

The dissemination of ARGs in the agroecosystem is a multifaceted process influenced by diverse factors, including horizontal gene transfer, microbial community structure, and environmental conditions [8], [20], [21]. The co-selection of resistance traits due to the widespread use of antimicrobials and the co-localization of ARGs on mobile genetic elements contribute to the rapid spread of resistance in agricultural settings [22]. Routine application of antibiotics in livestock farming and plant agriculture exerts selection pressures on microbial communities, fostering the emergence and proliferation of ARB [23], [24]. These ARB persist in soil, water, and crops, creating reservoirs of resistance genes. The transfer of resistant genes between environmental bacteria and potential human pathogens exacerbates the risk of antibiotic-resistant infections [25], [26]. This interconnected cycle of antimicrobial use, resistance development, and dissemination in agroecosystems underscores the urgent need for sustainable agricultural practices and stringent antibiotic stewardship to mitigate associated ecological and human health risks.

Developing effective strategies to limit the spread of resistance genes and mitigate the long-term impacts on human health and the environment is essential. As the global community confronts the challenges of AMR in agroecosystems, finding sustainable, science-driven solutions become imperative. Moving forward, a comprehensive and interdisciplinary approach that integrates microbiology, ecology, agronomy, and public health is required [27]. Therefore, this review aimed to synthesize existing knowledge on the interactive impacts of bioactive chemicals and antimicrobials in agroecosystems, providing a foundation for informed decision-making and targeted interventions.

In the following sections, we will examine the mechanisms that drive the interactive effects of antimicrobials and bioactive chemicals on AMR, analyze the dynamics of ARG spread within agroecosystem, and discuss potential strategies to address this escalating crisis. By unraveling the complexities of AMR in agricultural settings, we aim to enhance the growing body of knowledge that supports evidence-based policies and practices, promoting a sustainable balance between agricultural productivity and environmental health.

## Methodology

This review was written according to the strict guidelines for Preferred Reporting Items for Systematic Reviews and Meta-Analyses (PRISMA) to ensure transparent and comprehensive reporting of the relevant literature. Related published studies on antimicrobials and bioactive chemicals in agroecosystems were retrieved from the following databases –PubMed, Scopus, and ScienceDirect, using relevant keywords and Boolean operators (AND or OR or NOT). The input strings (“bioactive chemicals” OR “antimicrobials” OR “antibiotic-resistant genes” OR “antibiotic-resistant bacteria”) AND (“agroecosystem” OR “agricultural soil”) NOT (“water systems”) were investigated using the abstract, keywords, and title. However, book chapters, posters, abstracts, and conference proceedings were disregarded during the search process.

### Data extraction and quality assessment

An initial screening of the titles and abstracts of the existing studies was performed to determine their relevance and ensure that the selected articles focused on the primary issue of the study. Duplicate studies were eliminated from an Excel file summarizing the papers across the various databases. Subsequently, the inclusion and exclusion criteria were applied to examine each manuscript. All non-English studies, reviews, and publications not directly addressing the key topics were excluded. The final selected papers were compiled, and pertinent information was retrieved, including first authorship, publication year, and the relationships between antimicrobials, antimicrobial resistance, and bioactive compounds.

### Data synthesis and analysis

The narrative synthesis approach was used in this study to discuss the interactive impact of antimicrobials and bioactive chemicals. The reliability and confidence of the collected knowledge was evaluated qualitatively even though there was no specific grading system.

### Reporting

**Fig. 1:**
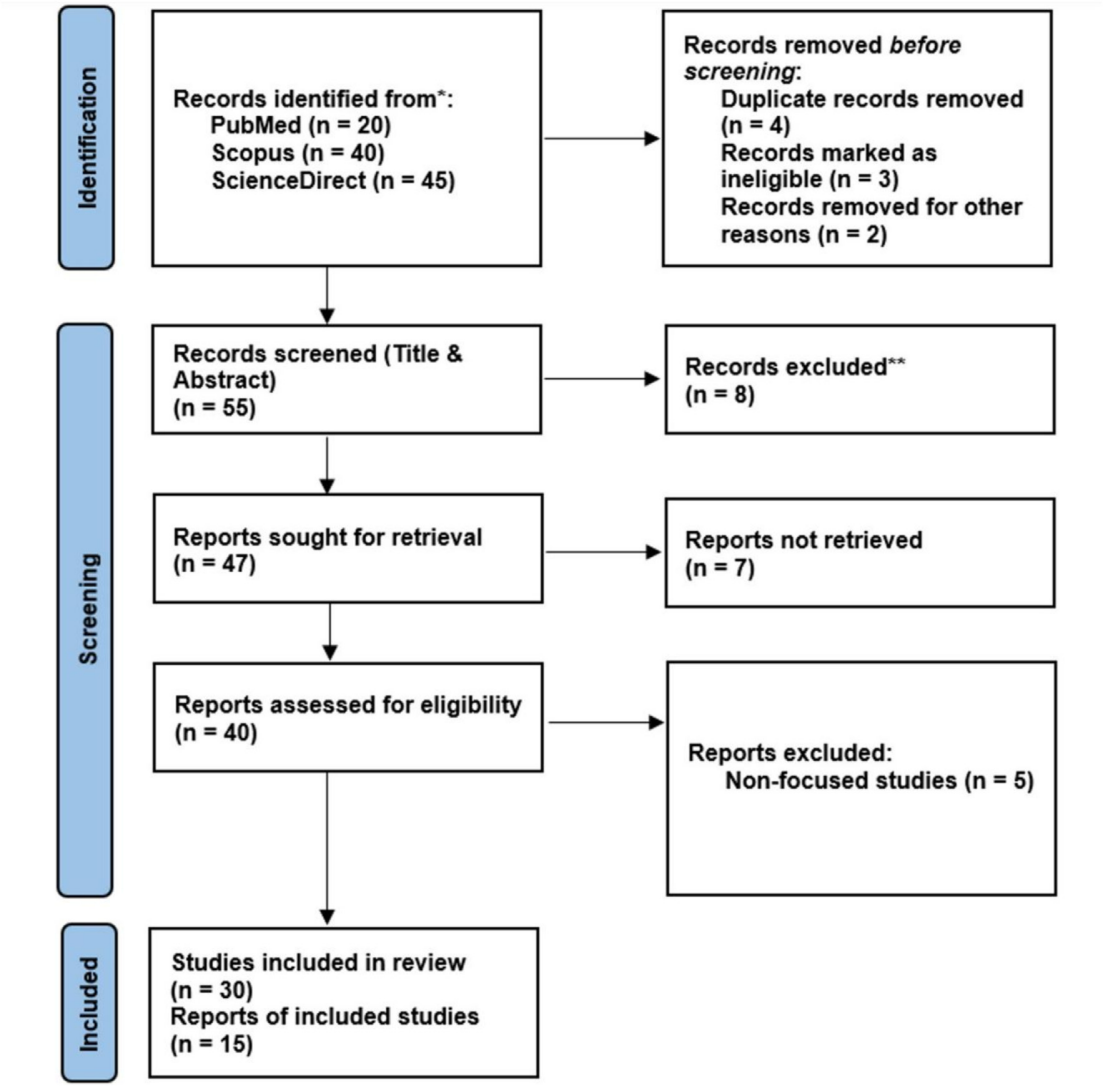
PRISMA 2020 process flow diagram of the study procedure.

**Fig. 2:**
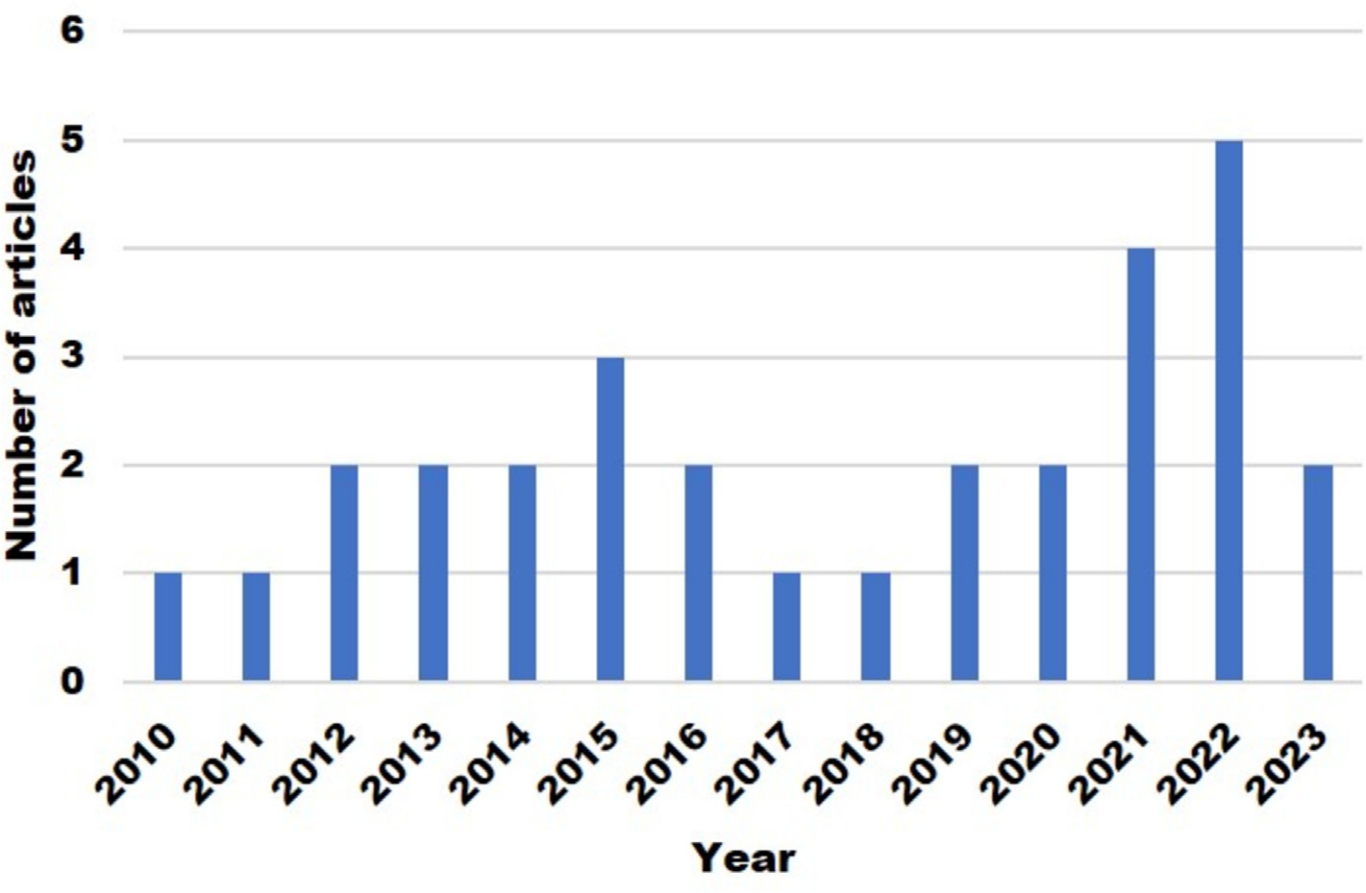
Total number of articles on antimicrobials and bioactive chemicals in agroecosystems gathered and published between 2010 and 2023.

Fifty-five (55) articles were garnered after scrutinizing the databases and removing replicas. Four documents—two non-English articles and two review documents—were removed after carefully examining the titles and abstracts. After a thorough final review of each manuscript, four additional papers were removed for being unrelated to the main topic of this review article. While all 47 publications were carefully examined, extra attention was given to papers published after 2010 received for their relevance. Ultimately, the review centers on 35 papers that provide an overview of antimicrobials within the agroecosystem and their interactive effects with bioactive chemicals on the dynamics of antimicrobial resistance (Fig. 1, Fig. 2).

### Occurrence and distribution of antibiotics and bioactive chemicals in agroecosystems

Contaminants of emerging concerns (CECs), such as antimicrobials, remain largely unmonitored under environmental legislation in many countries, making them an important global issue. Their widespread use in human and veterinary medicine has resulted in their presence across various environmental compartments, creating a transboundary environmental issue [28]. Most of the reported antimicrobial residues in natural environmental compartments, are bioactive chemicals potentially hazardous to soil bacteria and other agroecosystem organisms [29], [30], [31]. However, the occurrence and distribution of these antimicrobial residues and bioactive chemicals in agroecosystems have not been fully documented, thus requiring comprehensive studies on their concentrations and fate worldwide. Table 1 summarizes the characteristics of the existing studies on antibiotic compounds and bioactive chemicals, in crops, soil, and animal farm manure.

**Table 1:** Global occurrence and distribution of common antimicrobials and bioactive chemicals in agroecosystems.

**Contaminant type**	**Residue**	**Matrix**	**Concentration**	**Country**	**References**
**Crops**					
Antimicrobials	Tetracycline, oxytetracycline, and chlortetracycline	Vegetables	0.39 μg/kg	China	[32]
	Tetracycline, oxytetracycline, chlortetracycline, doxycycline sulfadiazine, sulfamethazine, sulfachlorpyridazine, and sulfamethoxypyridazine	Crop tissues	Not available	Spain	[33]
	Quinolone, macrolide, sulfonamides, β-lactams, nitroimidazole, and tetracycline	Lettuce	Up to 0.2 μg/L	Ghana	[34]
Bioactive chemicals	Acaricides, fungicides and insecticides	Greenhouse-grown chinese cabbage, cauliflower, Chinese chive, tomato, cucumber, and pakchoi crops	< 3400.2 μg/kg	China	[35]
	Organochlorines, organophosphates, herbicides, and fungicides	Horticulture	Up to 294 μg/L	Australia	[36]
	Herbicides, fungicides, and insecticides	Vineyard (Portugal), potatoes, onions, and sugarbeet (Netherlands)	0.003 ng/m^3^ and 10 ng/m^3^	Portugal and Netherlands	[37]
	Herbicides and insecticides	Genetically modified crops	Not available	USA	[38]
	Carbofuran, α-endosulfan, β-endosulfan, fenvalerate, malathion, and chlorpyrifos	Cauliflower, green chili peppers, eggplant, tomato, peas, bitter gourd, spinach and apple gourd crops	0.01–0.39 and 0.05–0.96 mg/kg	Pakistan	[39]
**Agricultural soil**					
Antimicrobials	Doxycycline, tetracyclines, oxytetracycline, ciprofloxacin, enrofloxacin, ofloxacin, norfloxacin, sulfamethazine, sulfamethoxazole, sulfadimethoxine, sulfadiazine, sulfamerazine, and sulfamethizole	Greenhouse and open-field soils	193 ng/g	China	[40]
	Tetracycline and sulfonamides	Agricultural soils	5.13–1628 μg/kg	China	[41]
	Tetracycline, pleuromutilin, and fluoroquinolone	Surface and deep soil layers	0.078 − 150 μg/kg dw	Spain	[42]
Bioactive chemicals	Herbicides and fungicides	Agricultural soils	2919.17 μg/kg	Argentina	[43]
	Glyphosate and aminomethylphosphonic acid	Topsoil samples from no-tillage fields	66.38 and 26.03 mg/kg	Brazil	[44]
	Herbicides	Cropland soils	1.01 − 1558.13 μg/kg	China	[45]
	48 fungicides, 25 insecticides and/or acaricides, 36 herbicides, and 2 safeners	Brownfields, woodlands, meadows, vineyards, orchards and arable lands	0.01 − 1115 ng/g	France	[46]
	Organophosphates, anthranilic diamide, neonicotinoid, benzimidazole, phenyl amide, and organochlorines	Agricultural soil samples	1.0 − 251 μg/kg	Nepal	[47]
	Insecticides, herbicides, and fungicides	Soil and air samples from agricultural land	Up to 63.6, 1.10, and 0.212 ng/g	South Africa	[48]
	Herbicides, fungicides, and insecticides	Organically managed agricultural soils	Up to 1170 μg/kg	Switzerland	[49]
**Animal farm manure**					
Antimicrobials	Sulfamethoxazole, trimethoprim, and sulfadimidine	Poultry manure samples	12.7–33.8 μg/g	Nigeria	[50]
	Fluoroquinolone, quinolone, lincosamide, macrolide, nitroimidazole, pyrimidine, sulfonamide and tetracycline	Poultry manure and soil samples	<1.0–392 μg/kg	Pakistan	[51]
	Enrofloxacin, ciprofloxacin, tylosin, sulfamethoxazole, and doxycycline	Poultry and bovine manure	0.4–153.5 ng/g	Poland	[52]
	Flumequine, ciprofloxacin, enrofloxacin, lincomycin, sulfadiazine, doxycycline, and oxytetracycline	Fattening calf slurry samples	31 − 10895 µg/kg	Belgium	[53]
	Cephalosporins, tetracyclines, penicillin, lincosamides, and trimethoprim-sulfonamide	Dairy farm	0.84–3.05 animal defined-daily doses /1000 cow-days	Canada	[54]
Bioactive chemicals	Estrogens (estrone, estriol, 17α-estradiol, and 17β-estradiol)	Cattle and pig slurry	54 − 244 ng/L and 138 − 861 ng/L	Switzerland	[55]
	Estrone, 17α-dihydroequilin,17α-estradiol, and 17β-estradiol	Animal manure and mushroom compost	Up to 462 ng/g	USA	[56]
	Estrone, estriol, α-zearalenol, testosterone, and 17β-estradiol	Runoff from manure amended row crop fields	100–200 ng/L	USA	[57]
	Estriol, estrone, testosterone, androstenedione, trendione, 17α- and 17β-estradiol, and 17α- and 17β-trenbolone	Anaerobic digesters	Up to 168 ng/L	USA	[58]
	Steroids	Swine farms	0.12 − 11200 ng/L	China	[59]

### Antimicrobials

**Fig. 3:**
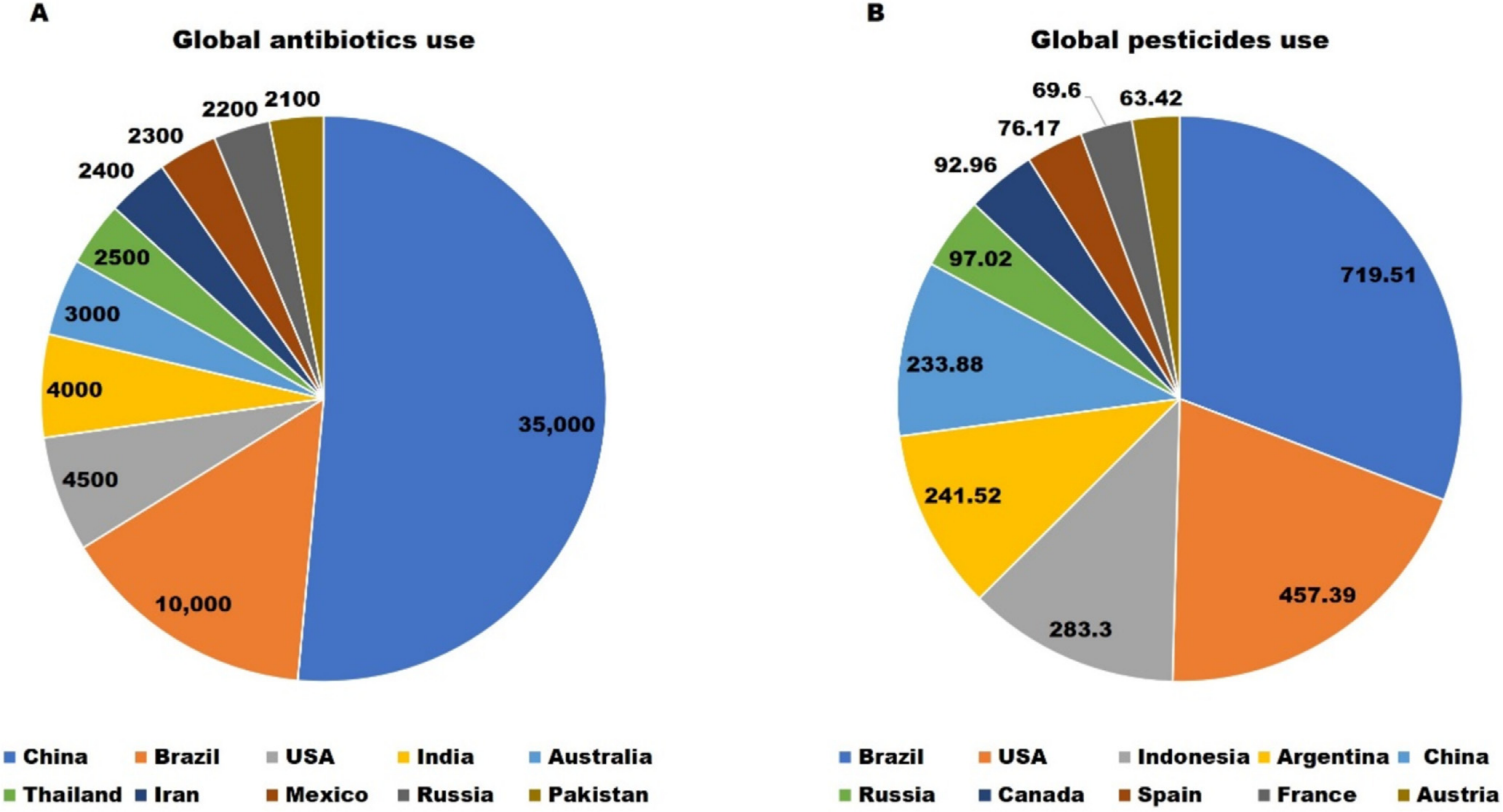
Global occurrence of antibiotics and pesticides in agroecosystems. The pie charts show (a) the estimated number of veterinary antibiotics used (in tonnes) in 2020 [62] and (b) the total agricultural consumption of pesticides (in metric tonnes) in leading countries in 2021 [74].

Antimicrobial occurrences in the agroecosystem raises significant concerns due to the evolution and emergence of ARB and the ecotoxicological behaviors these compounds can have on plants and animals [60]. The yearly global usage of antimicrobials, (veterinary and medicinal antibiotics), is projected to reach 100,000–200,000 tonnes by 2030 [61], [62]. In 2020, the highest consumers of antibiotics included China, Brazil, the United States, India, Australia, Thailand, Iran, Mexico, Russia, and Pakistan (Fig. 3A) [62]. Despite the significant presence of antibiotics in agroecosystems, only few studies have reported on antibiotic residues in livestock farms [63]. Notably, China is the world's largest producer and consumer of antimicrobials, with a significant portion of veterinary antibiotics being used in agriculture [64]. Veterinary antibiotics comprise the bulk of the overall amount utilized, particularly in agroecosystems. For instance, in the USA, they makeup around 70 % of the entire consumption, and about 70 % of those uses are not medical. Large-scale use of antibiotics in animal production is becoming widely adopted worldwide as drugs, particularly in the European Union, USA, China, and Southeast Asia, as feed additives or for other purposes (therapeutic, metaphylactic, and prophylactic purposes). However, it should be noted that Korea stopped using growth promotion antibiotics feed additives by 2012 and the European Union banned the use of antibiotics in feed for livestock production in 1998 [8]. The increased level of AMR in the agroecosystems results to a large extent, from the misuse/overuse of veterinary antibiotics in animal production and the ensuing manure applications to the land [65]. For example, veterinary antibiotics, such as tetracycline, recorded significantly higher concentrations (177.64 µg kg − 1 dry weight (dw)) in soil samples taken from a swine’s manure composting facility near a popular Korean agroecosystem [66]. According to Ho et al. [60], significant concentrations of flumequine (1331 µg kg^−1^ dw), the maximum concentration of doxycycline in broiler manure sample was 78516 µg kg^−1^ dw, and leftover doxycycline and enrofloxacin were found in every soil sample treated with manure from dung-amended soil, in Malaysia.

Furthermore, the three main antibiotics given sub-therapeutically to beef and dairy cattle in North America are sulfamethoxazole (9 %), tylosin (10 %), and chlortetracycline (16 %). These antibiotics have relatively high excretion rates: around 90 % of sulfamethoxazole, 65 % of chlortetracycline 50–100 % of tylosin are excreted by beef and dairy cattle [67]. Zhou et al. [63] reported thirty-two typical veterinary antibiotics in dung and manure-based fertilizers in Jiangsu Province, China. According to the authors, sulfamethazine and tetracycline were found in high concentrations in most manure and fertilizer samples, reaching up to 5650 and 1920 μg·kg^−1^, respectively [63]. Even though research on antimicrobials has advanced globally, relatively little knowledge on the abundance of these contaminants in agroecosystems is known.

### Bioactive chemicals

Bioactive chemicals are essential to contemporary agriculture in managing livestock, poultry, pests, diseases, and weeds. However, their extensive use to increase agricultural productivity can adversely affect both the environment and public health. One of the primary causes contributing to contamination in agroecosystems is the ineffective and uncontrolled application of these substances [68]. Despite this, the potential presence of bioactive chemical residues in untreated agricultural soils has received limited attention, with only a few recent studies have reported the occurrence of pesticides, steroids, and hormones, as major bioactive contaminants in agroecosystems [69]. For example, pesticide use has climbed by more than 40 % over the last 20 years, with over 2 million tonnes of pesticides used worldwide, China being the most significant contributor, and the United States and Argentina coming in second and third, respectively [70]. However, worldwide pesticide use is predicted to rise to 3.5 million tonnes by 2020 and beyond [70]. In 2021, herbicide occurrence reached 1.7 million metric tonnes globally, whereas the occurrence of other pesticide categories was less than one million (Fig. 3B). Because pesticides contaminate natural resources, their extensive and intensive use poses various health and environmental risks even while they increase crop productivity [49]. According to Debler [37], the calculated daily inhalation rates for individual pesticides and pesticide mixtures in two European agricultural areas (Portugal and Netherlands), were far below the Acceptable Daily Intake (ADI) with a margin of exposure (MOE) of > 1000 for the highest calculated daily inhalation rate for a child. Various pesticides residues such as myclobutanil, alachlor, buprofezin, bifenthrin, malathion, cypermethrin, fenpropidin, ethofumesate, trifloxystrobine, lambda, cyhalothrin, tebufenpyrad were found to contaminate an orchard within an agroecosystem located in Lebanon. According to the authors, the pesticides belonging to the insecticide and fungicide classes, including cypermethrin and myclobutanil, were mainly abundant, with a concentration of 1184.94 ng/g and 2241.08 ng/g, respectively [71].

Another study conducted in Macaronesia between 2018 and 2020 analyzed 139 agricultural soil samples for 218 pesticides, 37 pharmacological active chemicals, and 6 anticoagulant rodenticides. According to the findings, pesticides—primarily fungicides and insecticides— were the most often found bioactive chemicals, with exceptionally high quantities of the recently used insecticide fenbutatin oxide (302.1 ng g^−1^) and DDT metabolites (149.5 ng g^−1^ and 16.6 ± 35.6 ng g^−1^) [72]. These results, which comprise the most significant number of contaminant residues examined in agroecosystems to date, emphasize the necessity of establishing maximum residue limits, promoting soil monitoring programs for these contaminants and farmer education on the appropriate use of chemicals in agriculture, which are not yet present at the local or continental levels.

In addition, different synthetic variants of bioactive chemicals have also been produced for hormone replacement therapy or contraception, with the most widely used being the synthetic estrogen 17α-ethynylestradiol [73]. Although most hormones are found naturally, large-scale livestock farms release concentrated amounts of these chemicals into the soil and water by applying solid byproducts or effluents. For example, Bevacqua et al. [73], found various concentrations of hormones, including 63.4  ng/g progesterone and 44.1  ng/g estrone, in chicken litter from animal feeding operations in the U.S. Likewise, progesterone was found in all of the broiler manure samples in agricultural soils in Malaysia, which ranged from 8 to 367  μg kg^− 1^ dw [60]. Even though hormone steroids have been found in several locations across the world, not much is known about their distribution, fate, and transport in various environmental compartments presently.

## Environmental fate and transport of antimicrobials and bioactive chemicals in agroecosystems

### Sources, fate, and transport of antimicrobials in agroecosystems

**Fig. 4:**
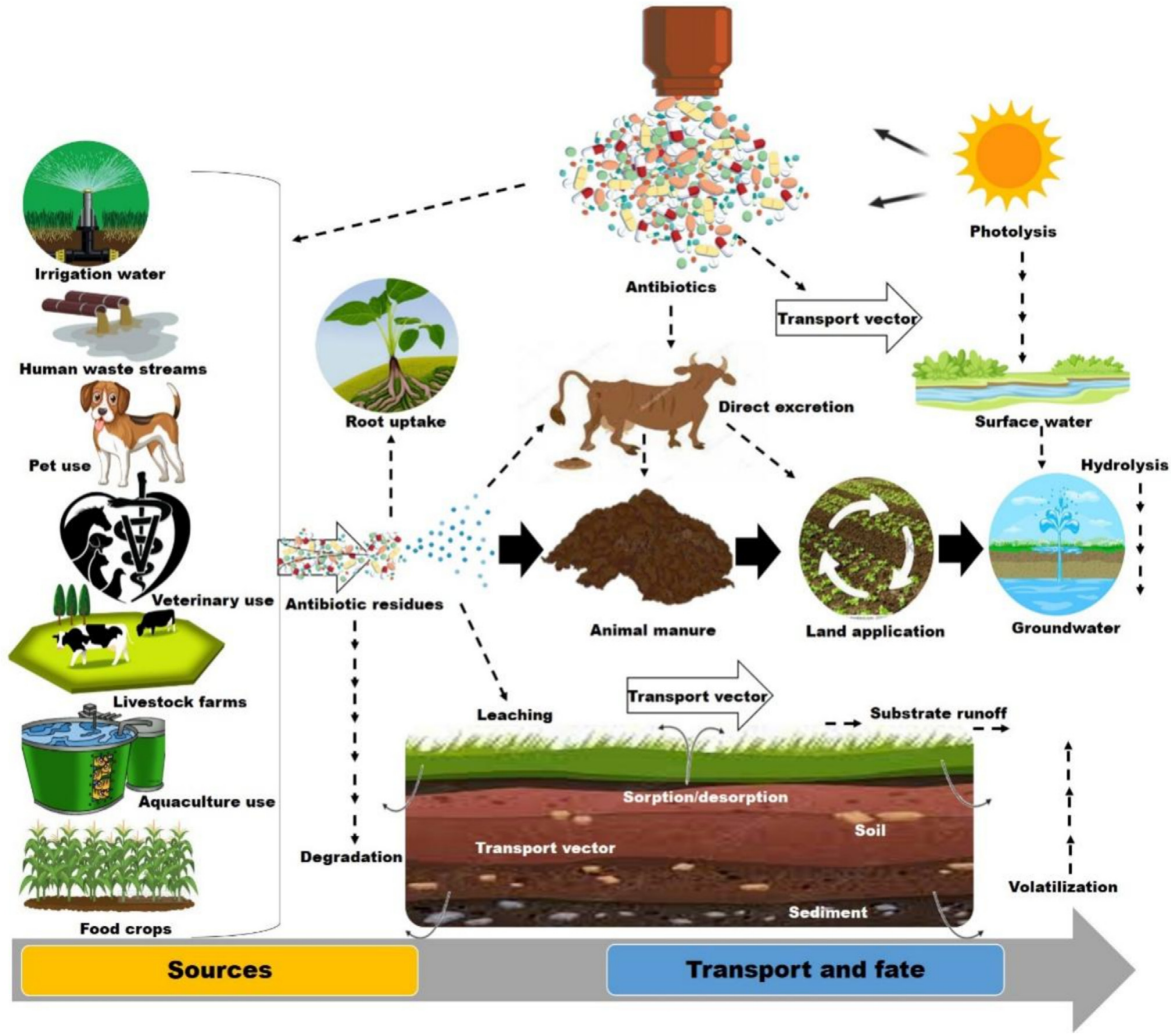
A schematic representation of the sources, transport, and fate of antimicrobials in agroecosystems. The release of antimicrobial residues used for various purposes, such as disease prevention and growth promotion in livestock and crop production, results in their infiltration into various environmental matrices, including soil, water, and crops, which consequently persist in soils and are transported through water systems, posing a significant risk to the ecological balance of agroecosystems.

Agroecosystems serve as a primary source of exposure to commonly used antibiotics, such as sulfonamides and tetracyclines, with additional contributions from human waste, veterinary waste, and livestock operations [75], [76]. Antibiotics can also enter these systems through groundwater or surface water contaminated by agricultural wastewater lagoons [8], [26]. Notably, animal manure, frequently used for soil amendment, is a significant source of antibiotic release due to its contamination with residues from antibiotics used for disease prevention, treatment, and growth enhancement in poultry and livestock. These antibiotic residues, which are not completely metabolized, are discharged unaltered into the soil, water, and manure, making the animal manure as a reservoir for resistance genes and antibiotic residues, regarded as contaminants with diverse environmental fates. This situation is exacerbated for antibiotics that are highly soluble in water, facilitating their rapid transport and ecotoxicity within agroecosystems [77]. The release of antibiotics occurs through several interconnected routes, including soil adsorption, degradation, transport via runoff and leaching, and plant uptake (Fig. 4). According to Du and Liu [31], significant release routes include fertilization with contaminated animal manures, biosolids, and sewage sludge, as well as irrigation with reclaimed water from contaminated sources such as sewage treatment plants and surface water. Active substances in the upper soil layer can accumulate or be absorbed by crops, while leaching and overland flow runoff can transport them into surface and groundwater [78]. Ultimately, the spatial–temporal dynamics and environmental fate of antibiotics in agroecosystems are influenced by human activities, crop management, water use, and soil microbes, which interact closely to drive these processes [79].

It has been reported that soil microbial community dynamics and microbial and enzymatic activity can be significantly altered during exposure to antibiotics present in manure. Several studies have also reported diminished plant growth, antibiotic buildup in plants, and inhibition of seed germination [80], [81], [82]. However, a comprehensive analysis of the fate and transport of antibiotics in agroecosystems is still lacking. A mass balance method was recently suggested following manure application to better understand the fate and transport of antibiotics in soil–plant systems [79]. The findings demonstrated that the soil–plant system recorded significant amounts of antibiotics after exposure to almost 98 % of the antibiotics in the applied manure fertilizer over 120 days. Less than 0.1 % of the antibiotics accumulated in the plants, but the majority (about 65 %) remained in the soil. The study suggested that rainfall-induced runoff, subsurface interflow, and soil water infiltration were additional routes via which antibiotics may be transported in soil–plant systems, although they did not contribute much [79].

Additionally, a long-term investigation was designed to discover more concerning the transport, behavior, and persistence of veterinary antibiotics in the subsurface of agricultural fields in northwestern Germany after manure application [83]. Thus, in an experimental field location, manure laced with a bromide tracer and antibiotics was applied at milligrams per liter, groundwater samples were taken systematically, and soil pore water was continuously extracted at varying depths to follow their route for two years—four of the seven target chemicals found in soil pore water leached into groundwater. With very few exceptions, the target chemicals' concentrations were in the nanograms per liter. Also, many antibiotics under investigation had already degraded or absorbed beneath the soil surface [83]. Furthermore, it was deduced from the data that long, warm, dry periods lead to attenuation of the antibiotics via increased soil absorption or degradation [83]. However, it is still unknown whether environmental antibiotic residues, bacteria resistant to antibiotics, and ARGs can directly enter or exist in the endophytic systems of exposed plants [84].

### Sources, fate, and transport of bioactive chemicals in agroecosystems

**Fig. 5:**
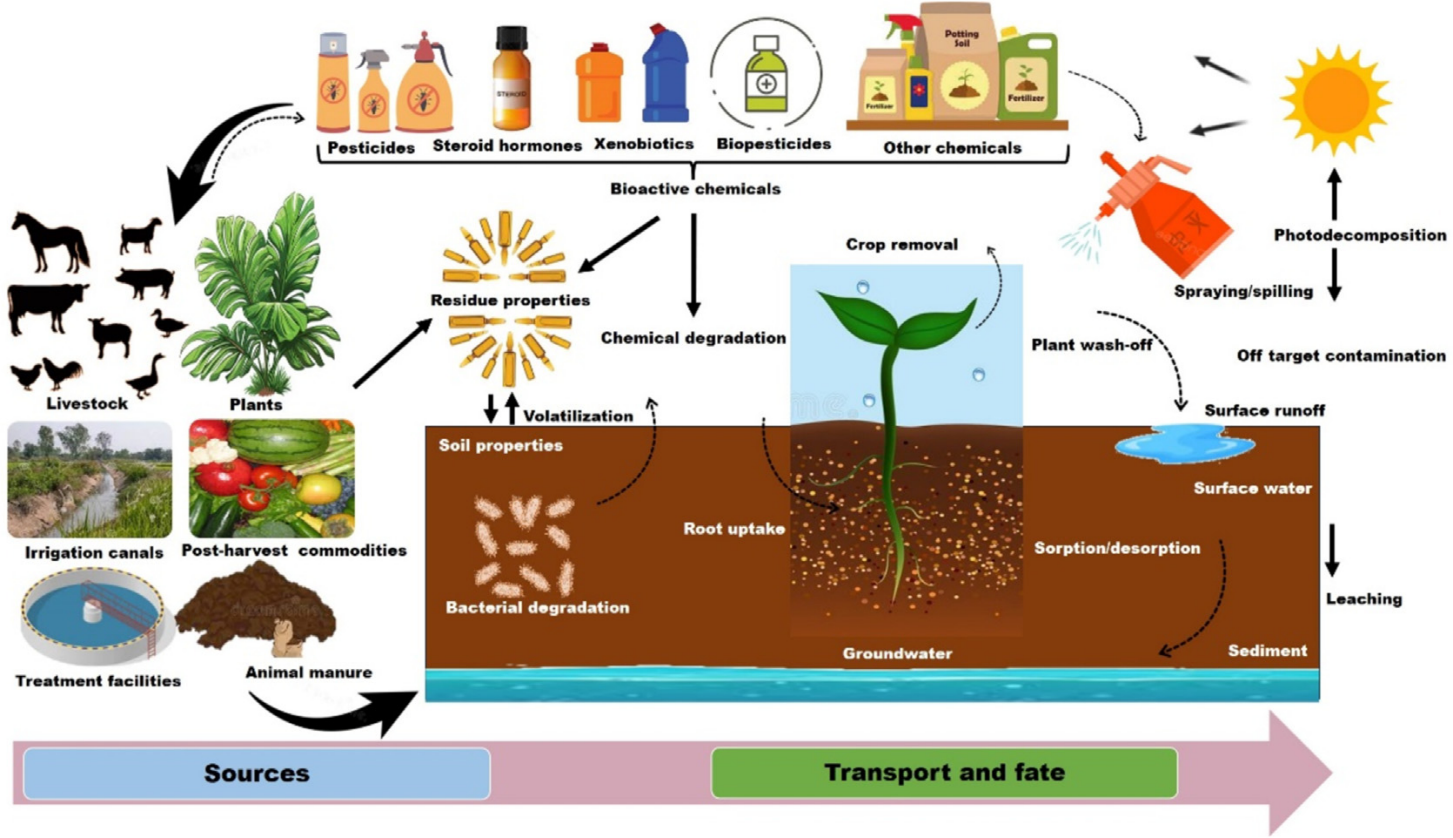
A schematic representation of the sources, transport, and fate of bioactive chemicals in agroecosystems. Bioactive chemicals used for pest control, weed management, and crop and animal improvement persist in agroecosystems. Agricultural activities, such as spray applications and soil amendments, introduce these chemical residues into the ecosystem. Various mechanisms, such as runoff, facilitate their transport while processes, including adsorption to soil particles, degradation, and leaching, influence their distribution within agroecosystems.

Bioactive chemicals are produced from various settings, including organisms, post-harvest commodities, treatment facilities, irrigation canals, livestock, animal manure, and plants. These substances include fumigants, herbicides, insecticides, fungicides, disinfection byproducts, and endocrine disruptors [85]. Much work has already been dedicated into defining the impact of concentration-dependent phenomena on the synthesis, degradation, transport, and toxicity of bioactive chemicals in agroecosystems [86]. Despite the well-established understanding that pesticide degradation through physicochemical processes, (bio)degradation, solid adsorption/desorption, and transport within and between agroecosystem compartments are key factors controlling their environmental fate (Fig. 5), the underlying mechanisms remain insufficiently understood to enable quantitative predictions of pesticide behavior across varying soil types and crops in agroecosystems. The challenges of soil's complex nature and lateral/vertical heterogeneity, including field-scale variability in space and time make the understanding of the fate of pesticides difficult [87]. The physical and chemical properties of pesticides (e.g., hydrophobicity and vapor pressure), soil characteristics (e.g., porosity, organic matter content, and microbial activity), environmental conditions (e.g., moisture and temperature), and land management practices (e.g., choice of cultivar, sowing date, and nitrogen fertilization) [88], all interact in a complex manner to determine the extent to which each mechanism contributes to the overall fate of pesticides [89].

Pesticides break down more quickly in soil through physiochemical processes and less quickly by biodegradation mechanisms, as the adsorption of chemical compounds onto soil and other solid materials, may have a catalytic role on the hydrolysis of chemicals [90]. At the same time, the fate and transport of pesticides in soil are largely dependent on the desorption process, which is the release of pesticide molecules after their adsorption onto solid particles, back into solution in response to a drop in soil solution concentration. The adsorption/desorption interaction determines if the solid adsorption phase provides a permanent sink or merely a transient reservoir of the pesticide [68]. The transport of pesticides from the top layer to the lower layers of the soil and downward in groundwater aquifers is known as leaching and is mainly controlled by water flow via the macropores in the soil, although the density and porosity of the soil also play a role. For instance, under similar environmental conditions, the herbicides benfluralin and propyzamide exhibit different leaching behaviors; this difference in leaching behavior is related to differences in the herbicides' water solubility rates [68]. A recent study demonstrated the adsorptive properties of different soils and leaching of pesticides to porosity [91]. According to their findings, different concentrations of chlorothalonil were found in two kinds of soils (clay and sandy soils were difficult to adsorb). Besides, under different applied concentrations, the residual amount of chlorothalonil in the leachate was significantly higher in topsoil (0–10 cm).

Notwithstanding the public health concerns of steroid hormones, little is known about the mechanisms and factors influencing the fate and transport of these chemicals. Due to their hydrophobic nature, the steroid hormones testosterone and 17 beta-estradiol were demonstrated to have a great propensity to be sorbed into the soil or sediment organic matter, while they break down quickly in water [92]. Consequently, it was shown that there was little chance of leaching into groundwater because these compounds persisted for a long time in plants and soil. For example, Chen et al. [93] hypothesized that plants may uptake residual steroid estrogens from the soil, which could endanger human health through food chains. This was demonstrated by using a wheat pot experiment to examine estrone and 17 beta-estradiol uptakes, and dissipation in various soil types. The findings showed that soils with greater levels of organic matter, silt, and clay inhibited the uptake of estrogens by plants. 17 beta-estradiol accumulated less in plants than estrone, primarily due to its shorter half-life and increased hydrophobicity. Instead of moving to the plant's shoots, estrogens are typically concentrated in the plant roots [93].

Furthermore, a 10–21 % boost in estrogen transport in the rhizosphere resulted from plant cultivation, increased enzyme activities, and the bacterial population. Besides, another study on the transport of steroid hormones, phytoestrogens, and estrogenic activity across a swine Lagoon/spray field system, reported that androgens and progesterone were inhibited, and conjugated hormones evaporated during storage in barn pits and the anaerobic lagoon. Estrone and equol persisted throughout the path of disposal of garbage [94]. However, after agricultural soils were treated with lagoon slurry, all analytes showed attenuation within two days, but androstenedione, progesterone, and equol were still persistent in the soil after two months of application [94].

The impact of steroid hormone contaminants on developmental and reproductive processes and neurological and immunological damage in mammalian and non-mammalian species are potential causes for concern. Because steroid hormones and their metabolites are not easily broken down, they survive and are physiologically active for a considerable amount of time, making the acute and long-term impacts of environmental exposure to these substances concerning [95]. Despite being banned in many developed countries, the three sex hormones (estrogen, testosterone, and progesterone) are naturally produced by mammals and birds, particularly during pregnancy, and can be discovered in significant concentrations in animal manure. Animal wastes can release hormones into the agroecosystem through surface runoff from agricultural land, where waste is applied as fertilizer, spills, direct discharge, or leaching from holding tanks, animal confinement areas, waste handling, and containment systems [96]. Estrogens, testosterone, and progesterone have all been found in runoff from fields treated with land-applied poultry litter. Moreover, although estrogen activity in storage lagoons can be measured, limited information is available regarding the fate of these steroids during composting [92].

Furthermore, it has been discovered that synthetic steroid hormones and their metabolites can be identified in cattle feces at significant concentrations and are persistent in the soil during the application of animal waste-derived compost. For example, Bartelt-Hunt et al. [97] assessed the fate of 16 steroid hormones and their metabolites during composting of beef cattle dung. The authors found that the levels of steroid hormones in cattle manure from beef cattle dropped by as much as 87 % after composting, although some specific steroids persisted during the study period. Additionally, a similar study reported the fate and transport of androgen, progestogen, and glucocorticoid residues during the composting of animal dung [96]. On the second day of composting, hormone concentrations increased significantly. Interestingly, steroids like methyltestosterone, melengestrol acetate, and hydroxyprogesterone caproate, which were absent in the initial compost, were later discovered. The authors suggested that the alterations in the microbial communities within the compost may have contributed on the dissipation of these steroid hormones. Compost-treated soils contained 12 steroids, while plant roots harbored 26, indicating a notable difference. These findings imply that hormonal residues in compost manure could lead to both plant uptake and soil contamination.

### Interactive impacts of antimicrobials and bioactive chemicals in agroecosystems

Simultaneous exposure to antimicrobials and bioactive chemicals is common in agroecosystems. Rather than a single antibiotic, various veterinary antibiotics, their metabolites, and other bioactive chemicals often combine to form antimicrobial residues [98], [99]. Although toxicological data are available for individual antibiotics, risk prediction and identification require understanding the impacts of these substances and their combinations on environmental receptors [100]. Unfortunately, very few studies have reported these chemical combinations' behavior, fate, transport, and ecological impacts in agroecosystems. Bioactive chemicals are employed in agriculture, where farm animals and pets that naturally produce sex hormones could receive prophylactic or therapeutic antibiotics exposed by spray drift or by strolling through treated fields. Most consumed antibiotics are not broken down and are combined with soil through manure, or compost to act as crop fertilizer, which may then be sprayed with bioactive chemicals such as herbicide on the spot [8]. For example, Kurenbach et al. [98] conducted a short-term study with different combinations of antibiotics and herbicides. Regardless of whether the non-antibiotic substance increased or decreased the antibiotic’s effectiveness, researchers found that acquired resistance occurred more frequently in certain conditions. This was especially evident when herbicides combinations were present, which made bacteria phenotypically resistant to higher concentrations of antibiotics. This is explained by how the herbicide affects an associated antibiotic's minimum selective concentration (MSC) or minimum inhibitory concentration (MIC). The MSC, which is lower than the MIC, is the lowest antibiotic concentration at which an individual's fitness varies due to the presence of the antibiotic. According to their findings, more environmental variables may impact bacterial competitiveness, which could improve an antibiotic's capacity to select for antimicrobial resistance. It is yet unclear, whether prolonged exposure to these pesticides at more environmentally relevant levels could encourage greater antimicrobial resistance [29].

**Fig. 6:**
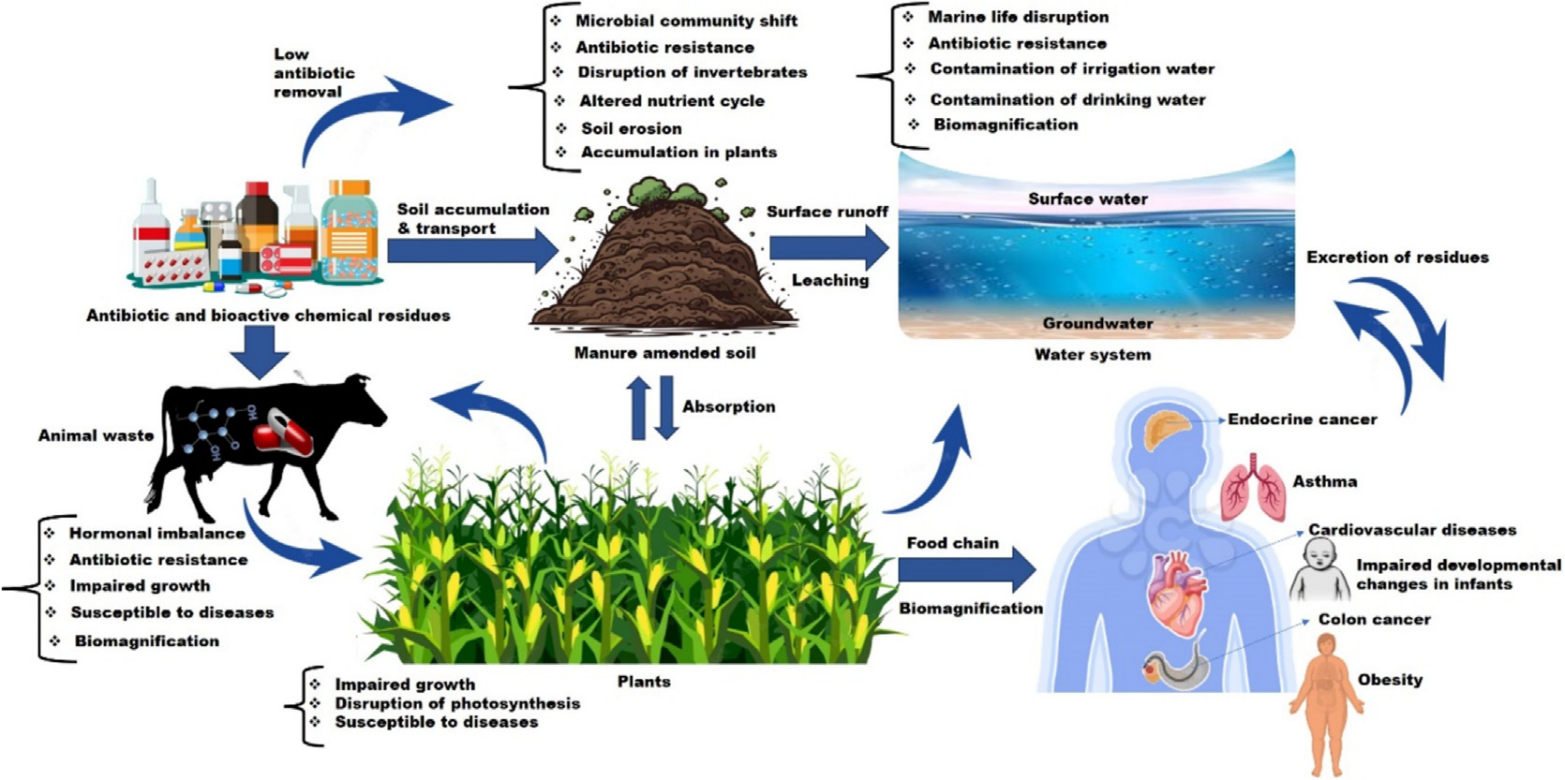
Mechanisms for the interactive impacts of antimicrobials and bioactive chemicals in agroecosystems. The coexistence of antimicrobials and bioactive chemicals, such as phytochemicals, hormones, and pesticides, poses a multifaceted challenge to agroecosystems. This emanates from agricultural practices that utilize antimicrobials for disease prevention and bioactive chemicals for crop enhancement and pest control, creating an environment where these compounds converge to impair ecological balance and public health due to low removal rates.

Elevated concentrations of hormones and antimicrobial residues have adverse effects on the agroecosystem, subsequently impacting public health by contributing to conditions such as obesity, endocrine cancer, colon cancer, cardiovascular endocrinology, and hypothyroidism (Fig. 6). This is attributed to these contaminants' hydrophilic, polar nature and low volatility. As a result, water movement and food chain dispersals will be the main ways they are biomagnified across the agroecosystem [101]. Li et al. [102] stated that the ecological environment may be threatened by the spread of estrogenic hormones and antibiotics from animal manures. The authors reiterated that these contaminants were widely persistent in animal manures at significantly higher levels and, based on the risk quotient assessment, could pose severe ecological threats to terrestrial organisms. Also, in a recent study by Hong et al. [103], manure-based organic fertilizer (MOF) samples were evaluated for 29 contaminants, including 20 antibiotic compounds and steroid hormones. The authors conclude that the combined effects of these contaminants would place plants at high risk, microbes and invertebrates at medium risk, and antimicrobial resistance selection in agricultural soils at significant risk. Also, the probable impacts of sulfamethoxazole and estrogens were investigated in animal waste [104], [105]. Although estrogens, such as estradiol or 17β-estradiol alone, significantly increased the bacterial strain diversity in the soil, their interaction with sulfamethoxazole as co-contaminants caused the beneficial effects of estrogens to shift and instead pose a severe threat to the native soil microbial population [105]. Consequently, in order to guarantee the safe application of animal manure on agricultural land, antibiotics and bioactive chemicals must be wholly eliminated from animal waste.

According to Jiang et al. [106], found that antibiotics, such as oxytetracycline, can significantly impact the breakdown of pesticides like atrazine in agricultural environments. Oxytetracycline prolonged atrazine's half-life by 1.27 and 1.59 times at concentrations of 5 mg/kg and 50 mg/kg, respectively, and altered its metabolite composition by affecting soil microorganisms. The study also showed a decrease in less harmful hydroxyl metabolites and an increase in more hazardous chloro-s-triazine metabolites, which are more likely to leach from the soil. This highlights the complex interactions between antibiotics and pesticides in agroecosystems.

Another example is seen in the study of Spolti et al. [107], who studied the sensitivity of 50 *Fusarium graminearum* isolates, including 3-acetyl deoxynivalenol and 15-acetyl deoxynivalenol trichothecene genotypes, to tebuconazole. Their findings showed that wheat plants inoculated with a tebuconazole-resistant isolate had significantly lower suppression of Fusarium head blight (FHB) and deoxynivalenol (DON) compared to those with a sensitive isolate, which is known for its aggressiveness and toxicity. However, metconazole treatment-maintained FHB and DON suppression in both isolates. Notably, after spraying a mixture of both isolates with tebuconazole, a higher number of tebuconazole-resistant isolates were recovered from the plants.

Furthermore, a recent study reported the potential public health impacts linked to high levels of antibiotics, hormones, and pesticides in U.S. cow milk samples [108]. The analysis compared residue levels of pesticides, antibiotics, and hormones (bovine growth hormone (bGH) and its associated insulin-like growth factor 1 (IGF-1)) in organic and conventional milk. Conventional milk showed pesticide and antibiotic residues exceeding federal limits, particularly sulfamethazine, amoxicillin, and sulfathiazole. Additionally, conventional milk had significantly higher concentrations of bGH and IGF-1, indicating the use of synthetic growth hormones. The study raises concerns about potential long-term health effects from these elevated hormone levels in conventionally produced milk.

## Ecological risks associated with bioactive chemicals, antimicrobials, antibiotic-resistant bacteria, and antibiotic-resistant genes in agroecosystems

Ecological risks related to emerging contaminants, such as antimicrobials, bioactive chemicals, ARB, and ARGs, include driving resistance, microbial community evolution and diversity, host-microbiome alteration, and ecosystem function variations [85], [109].

### Driving resistance

Bioactive chemicals and antimicrobials enhance bacterial antimicrobial resistance, promoting the spread of ARGs and ARB distribution. This occurs through various processes, such as the formation of gene mutations, the activation of efflux pumps, and the suppression of antibiotic-resistant outer membrane pores. For example, one crucial way pesticides affect the spread of ARGs in bacteria is by horizontal gene transfer (HGT). By promoting cell membrane permeability and raising the percentage of bacterial mobile gene elements (MGEs)—which aid in disseminating ARGs—pesticides aid in the conjugative transfer of ARGs [4]. In a recent study conducted by Liao et al. [110] in agricultural soils spanning 11 provinces in China, the application of herbicides and the presence of glyphosate residues in soils were correlated with elevated abundances of ARGs and MGEs compared to control sites without herbicide use.

Under natural conditions, antibiotics would hardly ever be encountered by bacteria in concentrations high enough to cause harm; therefore, there would be little chance for ARGs to arise and proliferate. This has suggested that antibiotic susceptibility in bacteria should be preserved by the prudent and modest usage of antibiotics, with most antibiotic exposures kept below the MIC [111]. However, limiting antibiotic usage to levels below MIC concentrations is far more challenging. Again, the bioactive chemicals' MIC has been assessed against multidrug-resistant pathogens, with values spanning from 0.4 to 1000 µg/mL. When combined with other bioactive compounds, triclosan has demonstrated effectiveness against antimicrobial resistance in microbial pathogens [26]. Sublethal concentrations of bioactive chemicals not only aid in the emergence of mutations that exert antimicrobial resistance but also mirror the selection of ARGs at sublethal concentrations of antibiotics. The coexistence of ARGs on the extrachromosomal genome enhances their resilience and facilitates transmission within pathogens or microbial communities. For instance, simultaneous resistance to bioactive chemicals and antibiotics results in an eight-fold increase in antibiotic tolerance for the ARB *S. aureus* compared to the sensitive wild-type strain. These genes are commonly found together on plasmids. Reports have recently highlighted the antimicrobial compound triclosan and the antidepressant agent fluoxetine in mediating resistance selection. The widespread presence of these compounds in various agro- and wastewater environments has raised significant concerns. Besides, aquatic ecosystem has been recognized as a reservoir for ARB and ARGs. For instance, Stange and Tiehm [112] detected ermB (42.1 %), tet(C) (40.8 %), sul2 (39.5 %), and sul1 (36.8 %), which code for resistance to macrolides, tetracycline and sulfonamides, respectively. According to the authors, these ARGs emanated from a combined sewer system, agricultural activities (resulting in water pollution by herbicides), and roadway runoff.

### Microbial community evolution and genetic diversity

Investigations into ARGs in soil have identified novel genes and enzymes responsible for bacterial genetic variations. Individual bacterial variations in physiology or genetic makeup can account for variations in antibiotic sensitivity [113]. Humans may experience the harmful effects of an antibiotic at various amounts due to the acquisition of genes or alleles via HGT or mutation, which results in a change in genotype [114]. Besides, organisms may differ intrinsically, for example, because of permeability variations [115]. HGT plays an essential role in ARG dissemination in agroecosystems. Conditionally expressed or repressed genes may also contribute to innate resistance, leading to a drop in intracellular antibiotic concentrations and an increase or decrease in antibiotic efflux or influx [116]. Different species may have different genes or expression induction thresholds, and individuals within a species may exhibit different phenotypes based on whether or not those genes were expressed prior to inhibition. According to Fernández and Hancock [116], this resistance resulting from altered gene expression is also referred to as an adaptive response, which modifies the phenotype of the bacteria in order to adapt them to their surroundings [117].

Furthermore, Xing et al. [14] reported that environmental pesticide exposure increased and diversified higher antimicrobial resistance in *Escherichia coli*. *E. coli* species exposed to 125 EC pesticides showed a 1.5-fold rise in the minimum inhibitory concentration (MIC) of streptomycin. Further analysis revealed that the *ftsI* gene mutated in cultures co-exposed to both pesticides and ampicillin. This gene encodes a protein that binds ampicillin, and the mutation likely altered the protein's structure, reducing ampicillin's binding affinity. This mutation may also lead to resistance against other beta-lactam antibiotics. SNP genotyping indicated that resistant populations from co-exposed cultures had the same mutation as those exposed to ampicillin alone, confirming that co-exposure triggered the mutation. These structural changes may hinder antibiotic entry into cells, thereby enhancing resistance [14].

### Ecosystem function alteration

Antimicrobials and bioactive chemicals in agroecosystems can induce various ecosystem and functional variations, contributing to ecological complexities and potential risks. These contaminants may selectively target specific bacteria, leading to shifts in the microbial population dynamics [29]. For example, antibiotics are designed to target specific bacteria, but their impact often extends beyond the intended pathogens. Also, the diversity of microbial functions in the agroecosystem is crucial for various ecosystem services, such as nutrient cycling, plant health, and disease suppression [13]. Antibiotics impact soil microorganisms, influencing their enzyme activity, capacity to metabolize diverse carbon sources, and overall microbial biomass. These effects extend to changes in the relative abundance of microbial groups, including Gram-negative bacteria, Gram-positive bacteria, and fungi [113]. Research employing nucleic acid analysis methods reveals that antibiotics alter the biodiversity of microbial communities, and agricultural and human activities influence the occurrence of various ARGs in soil. A total of 177 naturally occurring ARGs were identified by Van Goethem et al. [118], predominantly associated with encoding single or multi-drug efflux pumps. Common resistance mechanisms included those targeting the inactivation of β-lactam antibiotics, chloramphenicol, and aminoglycosides. The majority of ARGs (71 %) were found in Gram-negative bacteria, while Gram-positive Actinobacteria and *Bacilli* (Firmicutes) accounted for a smaller proportion (9 %), aligning with the taxonomic composition of the soils. Notably, there was a significant negative correlation between the abundance of ARGs per sample and species richness. This finding, combined with the absence of MGEs flanking ARGs, suggests that these genes are ancient acquisitions resulting from horizontal transfer events.

According to several reports, bioactive chemicals such as pesticides negatively impact bacterial biomass and community structure of agroecosystems, which may increase the likelihood of bacterial resistance to commonly used antibiotics [119], [120], [121]. For instance, after being exposed to triclosan in sludge samples, bacteria resistant to acidic environments were identified, and these microbes showed a selective effect on specific ARGs [122]. In addition, Cui et al. [123] found that low concentrations of ciprofloxacin (1 mg kg − 1) stimulated potential nitrification rates and significantly reduced microbial biomass without impacting soil respiration at higher doses. Also, the ciprofloxacin addition decreased the bacteria-to-fungi ratio and increased the Gram-positive-to-Gram-negative bacteria ratio. These findings confirm that a single application of ciprofloxacin can alter soil microbial communities' structure and function.

### Host-microbiome alteration

Exposure to emerging contaminants such as antimicrobials and bioactive chemicals is believed to adversely affect their hosts by disrupting the growth cycle and diminishing fertility. The impacts of pesticides on the rhizosphere microbiome of crop plants and the gut microbiome of pollinator insects, specifically managed populations of the western honeybee, *Apis mellifera* have been reported. The analysis establishes clear connections between pesticide mode of action and host-specific microbiome functionalities, particularly in relation to potential antimicrobial risks. For instance, the inherent differences in nitrogen metabolism within plant- and insect-associated microbiomes may determine whether neonicotinoid-based insecticides ultimately exhibit antimicrobial effects. Various context-dependent scenarios are explored, and beyond direct effects like the microbicidal action of the parent compound or breakdown metabolites, pesticides may indirectly influence the trajectory of host-microbiome coevolution in honeybees. This modulation extends to social behaviors and the insect gut-brain axis, potentially impacting plant-pollinator symbiosis [124].

According to Jakobsson et al. [125], antibiotics can influence host organisms by modifying microbial communities linked to animal hosts. Their investigations explored the short- and long-term effects of administering clarithromycin and metronidazole, a typical antibiotic regimen for *Helicobacter pylori*, on the native microbiota in the lower intestine and the pharynx. They found that the microbial communities of the participants in the untreated control group remained relatively stable. However, a week after starting antibiotic therapy, significant alterations with reduced bacterial diversity were observed in all treated patients in both locations. Additionally, they discovered that the microbiota of each subject responded differently to the antibiotic medication; a number of general tendencies were evident, including a significant reduction in Actinobacteria in the pharynx and feces immediately following treatment. Even though the diversity of the microbiota gradually returned to pre-treatment levels, the microbiota remained altered in some cases for up to four years following treatment. Furthermore, four years after treatment, significant levels of the macrolide resistance gene *ermB* were found, indicating that antimicrobial resistance can last longer than previously suggested.

In addition, the significant impact of bioactive chemicals on ecosystem health, including the induction of potentially life-threatening gene mutations, has been studied. As a substitute for the widely used endocrine-disrupting chemical, Bisphenol AF (BPAF) is recognized as a teratogen with developmental toxicities, posing potential ecological risks. For example, Wu et al. [126] unraveled the harmful effects of BPAF on spermatogenesis and spermiotiliosis in sexually mature mice exposed to BPAF (5, 20, 50 mg/kg/d) for 28 consecutive days. BPAF exposure significantly compromises the integrity of the blood-testis barrier and the quantity and quality of sperm in a dose-dependent manner. Sperm from BPAF-exposed mice exhibit severe DNA damage, altered SUMOylation and ubiquitination dynamics, and disrupted epigenetic inheritance with hypermethylation of H3K27me3, presumably due to the accumulation of cellular reactive oxygen species (ROS). Moreover, BPAF treatment impairs cytoskeleton architecture and tight junction permeability in primary cultured Sertoli cells, indicating dysfunction of actin regulatory proteins through the activation of ERK signaling, thereby disturbing the specialized microenvironment created by Sertoli cells for spermatogenesis.

Recently, changes in bacterial communities and ARGs in the gut of the model soil annelid species *Enchytraeus crypticus* and soil were investigated by Jin et al. [127]. The findings suggest that exposure to cypermethrin increased *Bacillus anthracis* pathogens in the gut microbiome of *E. crypticus* and the soil, significantly altering the latter's microbiome structure and even interfering with immune system functions. The coexistence of MGEs, ARGs, and potential pathogens, *Acinetobacter baumannii,* indicated the pathogens' elevated risk of pathogenicity and antimicrobial resistance. Also, the core-to-non-core bacterial abundance ratio contributed to the spread of MGEs and ARGs. Together, these findings offer a comprehensive understanding of cypermethrin's hitherto underestimated environmental risk on the spread of ARGs in the soil and non-target soil fauna. Although few studies have explicated the ecological risks associated with pesticides and ARGs, not much is known about the environmental risks related to the interactive exposure of other bioactive chemicals in agroecosystems.

## Analytical detection of commonly used antimicrobials and bioactive chemicals in agroecosystems

The development of analytical techniques over the past decade has improved the detection of numerous emerging contaminants of environmental concern, such as pesticides, antimicrobials, and steroid hormones (Table 2). One of the best ways to ensure appropriate traceability, early warning, and control of these compounds is to precisely and efficiently monitor the residual levels of these substances in the environment to protect ecological and environmental health [128]. Several varied and sensitive analytical techniques have been reported to detect these compounds in agroecosystems [129], [130], [131].

**Table 2:** Detection of commonly used antimicrobials and bioactive chemicals in agroecosystems.

**Contaminant type**	**Residue**	**Sample**	**Detection methods**	**Limit of detection**	**References**
Antimicrobials	Tetracycline, oxytetracycline and penicillin-G	Livestock	LC/MS/MS, and LC-ESI-QqQ	1.36, 1.66, and 2.89 µg/kg	[142]
	Tetracyclines, macrolides, and sulfonamides	Soil	HPLC-MS/MS	0.01 − 2.00 μg/kg	[143]
	Fluoroquinolone, quinolone, lincosamide, macrolide, nitroimidazole, pyrimidine, sulfonamide and tetracycline	Soil and poultry manure	LC-MS/MS	3 μg/kg	[51]
	Flumequine, ciprofloxacin, enrofloxacin, lincomycin, doxycycline, and oxytetracycline	Calf slurry and calf farmyard manure	UHPLC-MS/MS	40 and 210 µg/kg	[53]
	Nitroimidazoles, lincosamides, quinolones, sulfonamides, inhibitors of dihydrofolate reductase, fluoroquinolones, macrolides, tetracyclines, pleuromutilin, antiparasitic, and anti-inflammatory drugs	Natural springs	UHPLC-QqLIT-MS	0.31 − 3.6 ng/L	[144]
	Sulfonamides, quinolones, tetracyclines, macrolides, thiamphenicol, and lincomycin	Soil from vegetable fields	HPLC-MS/MS	0.1 − 20.0 μg/kg	[145]
	Sulfamethoxazole	Soil amended with cattle manure	HPLC-UV, LC-MS/MS, and ESI	0.3 and 0.4 μg/kg	[146]
Bioactive chemicals	Tricyclazole (fungicide), butachlor (herbicide), and clothianidin (insecticide)	Rice fields	GC-ECD andHPLC	0.005 mg/L	[147]
	Phytochemicals	Leaves	MALDI-HRMS	Not available	[148]
	Pesticides	Soil	HPLC-MS/MS	0.01 − 2.0 μg/kg	[143]
	Hormones	Cattle and pig slurry	ESI-LC-MS/MS	1.3 − 6.3 ng/L	[55]
	Pesticides	Wheat farm	LC-MS/MS	0.037 and 36 μg/kg	[149]
	Hormones	Crop fields	SPE-LC-MS/MS	Not available	[57]
	Pesticides	Vegetables	LC-MS/MS and GC–MS/MS	1.0 − 2.5 µg/kg	[150]
	Hormones	Animal manure, mushroom compost, and biosolids	SPE-GC/MS	Not available	[56]
	Pesticides	Cropland soils	HPLC-MS/MS coupled with GC–MS/MS	0.01 − 0.30 ng/L	[45]
	Pesticides	Vegetables	GC-MSD	0.01 − 0.07 mg/kg	[39]
	Steroids	Swine farms	SPE-ESI-UHPLC-MS/MS	Not available	[59]
	Pesticides	Chinese vegetables	GC–MS and UHPLC-MS/MS	0.001 − 0.005 mg/kg	[35]
	Organophosphorus pesticides	Soil	USL-SPE/DSLLME	0.012 and 0.2 ng/g	[151]

DSLLME, dispersive-solidification liquid–liquid microextraction; ECD, electron capture detector; ESI, electrospray ionization; G.C., gas chromatography; GC–MSD, gas chromatography coupled with a mass selective detector; GC–MS/MS, gas chromatography triple quadrupole mass spectrometry; HPLC-UV, high-performance liquid chromatography coupled to an ultraviolet detector; HPLC, high-performance liquid chromatography; LC-MS/MS, liquid chromatography coupled with tandem quadrupole mass spectrometry; LC- ESI-QqQ, liquid chromatography coupled with triple quadrupole mass spectrometer; MALDI-HRMS, matrix-assisted laser desorption/ionization high-resolution mass spectrometry; MRM, multiple reaction monitoring; SPE, solid phase extraction; UHPLC-MS/MS, ultra-high-performance liquid chromatography tandem mass spectrometry; UHPLC-QqLIT-MS, ultra-high-performance-liquid chromatography coupled to a quadrupole-linear ion trap tandem mass spectrometer; USL-SPE, ultrasound leaching-solid phase extraction.

Extraction methods such as ultrasound-assisted extraction (UAE), microwave-assisted extraction (MAE), pressurized liquid extraction (PLE), and QuEChERS (Quick, Easy, Cheap, Effective, Rugged, and Safe) have been reported for the determination of antibiotic residues in agroecosystems [1]. Solid-phase extraction (SPE) is frequently used to clean up extracts before they are analyzed using liquid chromatography-tandem mass spectrometry (LC-MS/MS) [1], [9], [132]. The detection of polar compounds with the use of LC-MS and of their transformation products (TPs) has gained increasing attention due to the availability of analytical technologies in the last years, that detect unknown compounds as well as known ones [133]. The high mass accuracy and resolution of the High-Resolution Mass Spectrometry (HRMS) technology recently, has also made possible the wide-scope, suspect and non-suspect screening of thousands of organic compounds and their transformation products, at trace levels [134]. Suspect screening has been widely established due to the availability of different suspect lists such as the NORMAN SusDat [135], [136], keeping in mind that ‘smart’ suspect screening within a suspect list must take place using an appropriate analytical method for compound identification.

Interestingly, a method for determining critically essential antibiotics (listed by the World Health Organization (WHO)) such as tetracycline, diaminopyridine, and sulfonamides was recently proposed. The process is based on LC-MS/MS analytical determination, dispersive solid-phase extraction clean-up, and ultrasound-assisted extraction. The technique was approved for use on agricultural soil and sewage sludge from various treatment phases. For most chemicals, the detection limits were between 0.03 and 7.50 ng/g dw. The range of accuracy values was 70–102 % with less than 17 % precision. When the technique was applied to actual samples, it was discovered that the antibiotic classes with the highest concentrations across all sample types were macrolides and fluoroquinolones. The lowest antibiotic concentrations were found in soil samples (maximum concentration: 93 ng g^−1^ dw, corresponding to a clarithromycin metabolite) and compost (highest concentration: 27 ng g^−1^ dw, corresponding to norfloxacin). The suggested technique is the first to be created to date for identifying multiclass antibiotics and their primary metabolites in sludge from various phases of treatment [1].

Recently, inner-filter effect (IFE)-based fluorescence assays have attracted attention for accurate, dependable, and cost-effective reporting of pesticides and antibiotics because they eliminate the laborious surface modification or labeling processes. As a result of the tremendous advancements in this field, the most recent developments in IFE-based fluorescence assays for detecting the residual levels of antibiotics and pesticides, such as nitro antibiotics, tetracyclines, carbamates, and nicotine, as well as organophosphates and chloronicotinoids have been thoroughly examined [128]. This technique also detected pesticide residues such as organophosphates, nicotine, chloronicotinoids, and carbamates from agricultural crops [137]. Also, using MAE followed by liquid chromatography-mass spectrometry (LC-MS) coupled with electrospray ionization (ESI) in positive mode, concentrations of several estradiol-mimicking chemicals, such as estriol, 17α-ethynylestradiol, and 17β-estradiol were detected in sewage sludge and biosolids. The technique yielded limits of detection (LODs) ranging from 0.6 to 3.5 μg·kg^−1^ [138].

Interestingly, a multi-residue analytical technique quantified one hormone and nine antibiotics in soil, manure compost, and broiler manure. The procedure was contingent on SPE and ultrasonic extraction and was later subjected to LC-MS/MS for instrumental analysis. The new technique demonstrated linearity over a concentration range from its experimental quantification limit (IQL) to 500 ng/mL, with correlation values greater than 0.999. The general performance of this technique was satisfactory for the bulk of the analytes, and the recoveries ranged from 63 % to 121 % across various sample matrices. The 10 target analytes in the soil, broiler manure, and manure compost samples had corresponding method quantification limits (MQLs) of 2–10, 3–16, and 5–15 μg/kg dw. Tilmicosin, an antibiotic documented for the first time in the environment, was also detected. The developed technique was then used to detect and quantify veterinary antibiotic and hormone residues in the terrestrial agroecosystems using broiler manure samples and amended agricultural soils [139]. Another study investigated the capacity of the QuEChERS extraction method followed by LC-HRMS for the detection of pesticides and their transformation products (TPs) in various soil samples with different agricultural applications in Switzerland [132]. The study investigated 80 polar pesticides and more than 90 TPs in topsoil samples collected from 1995 to 2008. 10–15 pesticides were identified in the majority of the sites (45 % detection rate) at concentrations of 1–330 μg/kg soil. Approximately 80 % of all applied pesticides on the soil samples could be detected, as parent compounds or as their associated TPs, showing their persistence for more than a decade. Chiaia-Hernández et al. [135] used LC-HRMS/MS and an in-house suspect list, for sample screening of 13 samples from the Swiss National Soil Monitoring Network and 3 sediment cores of an urban/agricultural use lake. The suspect screening included > 500 halogenated compounds. The presence of 96 compounds was confirmed with 34 compounds common in soil and sediment, including pesticides, antimicrobials and personal care product compounds.

Furthermore, screening and confirmatory analytical techniques have been employed recently to analyze hormonal residues in agroecosystems. This is pertinent in order to monitor the higher stability and longer environmental persistence exhibited by synthetic hormones in farms and poultry. The occurrence of 34 natural or synthetic hormones including progestins, androgens and estrogens was investigated in 430 samples of soils of agricultural greenhouses and open fields cultivating various vegetables, by Yang et al. [140]. An LC-MS/MS system was used with recoveries of the compounds ranging from 82 % to 115 %, a method detection limit (MDL) of 0.006 ng/g-0.108 ng/g and method quantification limit (MQL) of 0.019 ng/g-0.360 ng/g. A widespread prevalence of the target hormones was established, with their detection in 99.3 % of the soil samples. Moreover, according to Qaid and Abdoun [141], it is possible to quickly detect hormonal drugs (bovine somatotropin, trenbolone acetate, progesterone, melengestrol acetate, estradiol, and zeranol) residues in meat using the following analytical techniques: high-performance thin-layer chromatography (HPTLC), LC-MS coupled with atmospheric pressure chemical ionization (APCI), and HPLC-ESI tandem mass spectrometry.

## Current technologies for the removal of antimicrobials and bioactive chemicals in agroecosystems

Antimicrobials and bioactive compounds in agroecosystems negatively affect the soil, water, and plants, among other components of the environment. Most of these contaminants are now considered a priority because of their bio-toxic qualities, necessitating quick response to remove them from contaminated environments [152]. To mitigate the effects of these contaminants, action must be taken, which includes various treatment and removal techniques employed for remediating antibiotics and bioactive compounds (Table 3). Several remediation techniques based on physical, chemical, and biological treatments are widely known to remove these contaminants from agroecosystems [85], [153], [154], [155], [156]. These treatment and removal technologies include biological treatment, such as bioremediation and phytoremediation; chemical treatment, such as advanced oxidation processes (AOPs) and activated carbon adsorption; and physicochemical treatment, such as membrane filtration and coagulation and precipitation. Other removal methods include activated sludge process, constructed wetlands, U.V. irradiation, ozonation, electrochemical treatment, ion exchange, and reverse osmosis [152], [157], [158]. However, the production of toxic sludge, insufficient removal, high cost, operational expenses, and the demand for trained personnel for operation and maintenance have all limited the sustainable adoption of these technologies.

**Table 3:** Removal efficiency of antimicrobial and bioactive chemical residues from agricultural and environmental samples using various treatment methods.

**Contaminant type**	**Residues**	**Sample**	**Removal method and conditions**	**Remarks**	**References**
Antimicrobials	Sulfadimidine, roxithromycin, doxycycline, tylosin, tetracycline, oxytetracycline, chlortetracycline, norfloxacin, enrofloxacin, ciprofloxacin, and sulfamethoxazole	Digested piggery wastewater	Biofilm membrane bioreactor; duration: 191 days; pH: 6–8; and dissolved oxygen: <5 mg L^−1^	86.8 % removal rate	[170]
	Fluoroquinolones, enrofloxacin (ENR), ciprofloxacin (CIP), and sulfonamide, sulfamethoxazole (SMX)	Manure and digestate from a biogas plant in a cattle farm	Phytoremediation with plant pots filled with 500 g cattle manure-amended soil	CIP and SMX removal up to 55–79 % and 70–85 %	[171]
	Tetracyclines, sulfonamides, tetracycline, and antibiotic-resistant genes, sul1 *and *sul2	Dairy manure	Advanced anaerobic digestion	Roughly 5 % and 10 % removal of *sul*1 and *sul*2 genes	[172]
	Tetracyclines, quinolones, and sulfonamides	Swine manure	Anaerobic digestion and composting on an industrial scale	> 97 % removal rate	[173]
	Doxycycline	Pig manure and digestate	Anaerobic digestion	61 % and 76 % removal rates in pig manure and digestate	[174]
	Sulfonamides	Swine manure	Composting process	The thermophilic aerobic composting technique effectively removed sulfonamides by reducing their half-lives to less than 3 days	[175]
	Veterinary antibiotics	Broiler manure	Composting process	After 40 days of composting, the manure had lost more than 99 % of the 9 antibiotics and 1 hormone	[176]
	28 multiple-class veterinary antibiotics	On-farm pig slurry	Batch reactor with a nitrification–denitrification process and solid–liquid separation	Tetracyclines and fluoroquinolones demonstrated intermediate removals (50 and 40 %), whereas lincomycin had the highest removal, reaching 100 %	[177]
Bioactive chemicals	Steroid hormones and pesticide residues	Agricultural wastewater	Membrane distillation-enzymatic membrane bioreactor	>90 % removal rate	[178]
	Organo-phosphorous pesticides	Soil	Three bacteria, *Pseudomonas aeruginosa*, *Enterobacter cloacae,* and *Enterobactor ludwigii* isolated from domestic sewage	Combined bacterial cultures were more effective at removing pesticides from soil and broth	[168]
	Diazinon	Aqueous solution	Coconut shell-modified biochar; adsorbent dose of 5.0 g/L chemically modified phosphoric acid; and pH 7	98.96 % diazinon removal	[179]
	Oxyfluorfen, tebuconazole, and metalaxyl	Agricultural soil	Vermicompost-based bioremediation platforms with 4 fungal strains and 6 bacterial derived from wine and olive oil wastes	7.7, 1.6, and 3.8-fold removal rates for oxyfluorfen, tebuconazole, and metalaxyl	[180]
	Estrone and 17β-estradiol	Manure-containing water	Vegetable oil capture procedure containing eight vegetable oils	94–98 % and 88–97 % removal rates for estrone and 17β-estradiol	[181]
	7β-estradiol	Waste samples such as manure-applied soil, composted manure, and poultry and livestock wastes	Bioremediation with bacterial co-culture of the genus of *Acinetobacter* and *Pseudomonas,* isolated from manure	>90 % removal rate	[182]
	Atrazine	Aqueous solutions	Submerged biological aerated filter	97.9 % and 98.9 % removal rates	[183]
	Dicofol	Aqueous solutions	Novel photocatalyst fabricated nanocomposite, MoS2/ZnS@NSC	Fabrication of pure nanocomposites and the outcomes of photocatalysispromote the complete removal of dicofol	[184]
	Estrogens	Livestock wastewaters	Constructed wetlands	90 % removal rate	[185]
	Acephate	Aqueous solutions	Novel green mesoporous MCM-41 nanocomposite and Co_3_O_4_ from silica gel extractedchemically from rice husk	Complete removal of various acephate concentrations (50 mg/L after 40 min), (100 mg/L after 60 min), (150 mg/L after 100 min), and (200 mg/L after 140 min)	[186]
	Fungicides	Grapes, pomaces, musts, and lees	Whitewine-making process involving the vinification of white grapes	90 % − 99 % removal rate	[187]

### Removal technologies for antimicrobials

It is imperative to discuss antimicrobial removal technologies thoroughly, given the wealth of knowledge on antimicrobial resistance and the current treatment technologies intended to remove antibiotics from agroecosystems efficiently. For example, nanofiltration was suggested to efficiently remove antibiotics from wastewater treatment plants (WWTP) effluent. Liu et al. [159] investigated the removal of four antibiotics, namely azithromycin, roxithromycin, ofloxacin, and norfloxacin (NOR), due to their high occurrence in the effluents from Dalian (China) wastewater treatment plants (WWTPs) using a combination of nanofiltration with ozone-based advanced oxidation processes. Antibiotic rejection rates exceeded 98 % in all nanofiltration trials. Nanofiltration concentrate underwent treatment through UV/O_3_ processing, ozonation, and UV_254_ photolysis. UV_254_ photolysis was found to be ineffective in degrading the four medications, while ozone-based methods exhibited efficient elimination within just 30 min. Notably, during UV/O3 treatment, a synergistic effect between O_3_ and UV contributed to removing antibiotics.

In addition, conventional removal methods, such as stand-alone constructed wetlands (CWs) and membrane bioreactor (MBR) systems, fall short of addressing environmental degradation [160]. CWs were recently proposed to remove antibiotics from agricultural wastewater. The removal of contaminants via natural processes, including photodegradation, adsorption, biodegradation, hydrolysis, volatilization, etc., is accomplished by the CWs system. However, wastewater parameters, substrate surface area, and the contaminants' physicochemical characteristics significantly impact CW performance [101]. An integrated Bioelectrochemical System (BES) has been developed to address this, incorporating Microbial Fuel Cells (MBR-MFC, CW-MFC-MEC, and CW-MFC) for antibiotic removal. Additionally, a unique continuous-flow MFC-sorption system has been established for tetracycline removal. It was observed that increased electrolyte concentration, elevated tetracycline concentration, and lower pH values could enhance tetracycline adsorption capability [161].

### Removal technologies for bioactive chemicals

Several studies have reported the current developments and progress in the use of various techniques for the possible removal of bioactive chemicals from agroecosystems [162], [163]. Recently, adsorption technologies have been used to remove pesticides and steroid hormones. High levels of steroid hormones such as estrone and progesterone were reported, with individual hormone levels reaching up to 1478 ng g^−1^ dw or 22.5 mg kg^−1^N in the biogas final digestion output that is used as fertilizer to croplands after anaerobic digestion. This showed that using the final digestate output on croplands leads to the release of hormones into the environment, although the biogas digestion procedure was unable to eliminate steroid hormones from cattle manure completely [164]. Even though the availability of adsorbents has been a major challenge of adsorption technologies, they are the most widely applicable technique for removing these contaminants because of their low cost, making them readily implemented in developing countries with limited access to capital, trained labor and advanced technology [157]. However, the adoption of hybrid removal techniques presents chances for developing novel contaminant removal strategies [165].

Interestingly, the eco-friendliness, cost-productivity, and low risk of secondary contaminant generation associated with bioremediation technology make it one of the safest ecological removal methods considered globally. However, the use of biological species remains critical in bioremediation techniques because of their strong natural flexibility and topographical circulation. Besides, the successful utilization of biological species must ideally involve indigenous species, which could facilitate decreased disturbance caused by invasive species [166]. This is because assessing the environmental risk of agricultural synthetic chemicals and guiding the advancement of their remediation depends on knowledge of how microorganisms proliferate in the presence of contaminants [167]. Biological species and adsorption techniques are currently adopted to efficiently remove bioactive chemical residues from the environment [168]. For example, pesticides found in the soil and water environment were eliminated using *Pseudomonas stutzeri*, isolated from the pesticide-contaminated crop area. Utilizing biomass from agricultural byproducts, specifically *Delonix regia* seeds, as a bioadsorbent, the pesticides were removed from soil and water. According to the authors, combining microbe-specific degradation pathways with *Delonix regia* seeds may be crucial in eliminating harmful and uncontrollable pesticides in the soil and water. The authors asserted that the blend bioadsorbent could remove chlorpyrifos (with a shoot-up removal reaching 95.29 %) from the pesticide-contaminated soil and water [169].

Gamma radiation has proven effective in significantly reducing pesticide levels in vegetable samples. As radiation doses increased, pesticide levels proportionally decreased. At a dose of 0.5 kGy, reductions of 35–43 %, 40–48 %, and 30–45 % were observed for chlorpyrifos, diazinon, and phosphamidon, respectively. When the radiation dose was increased to 1.0 kGy, these reductions reached 80–91 %, 85–90 %, and 90–95 %, respectively. The authors concluded that gamma radiation at 1.0 kGy effectively removed 80–95 % of certain pesticides found in high concentrations. Although laboratory studies have demonstrated success in removing such contaminants, further field research is needed to obtain more accurate data and results [137].

## Conclusion and future perspectives

**Fig. 7:**
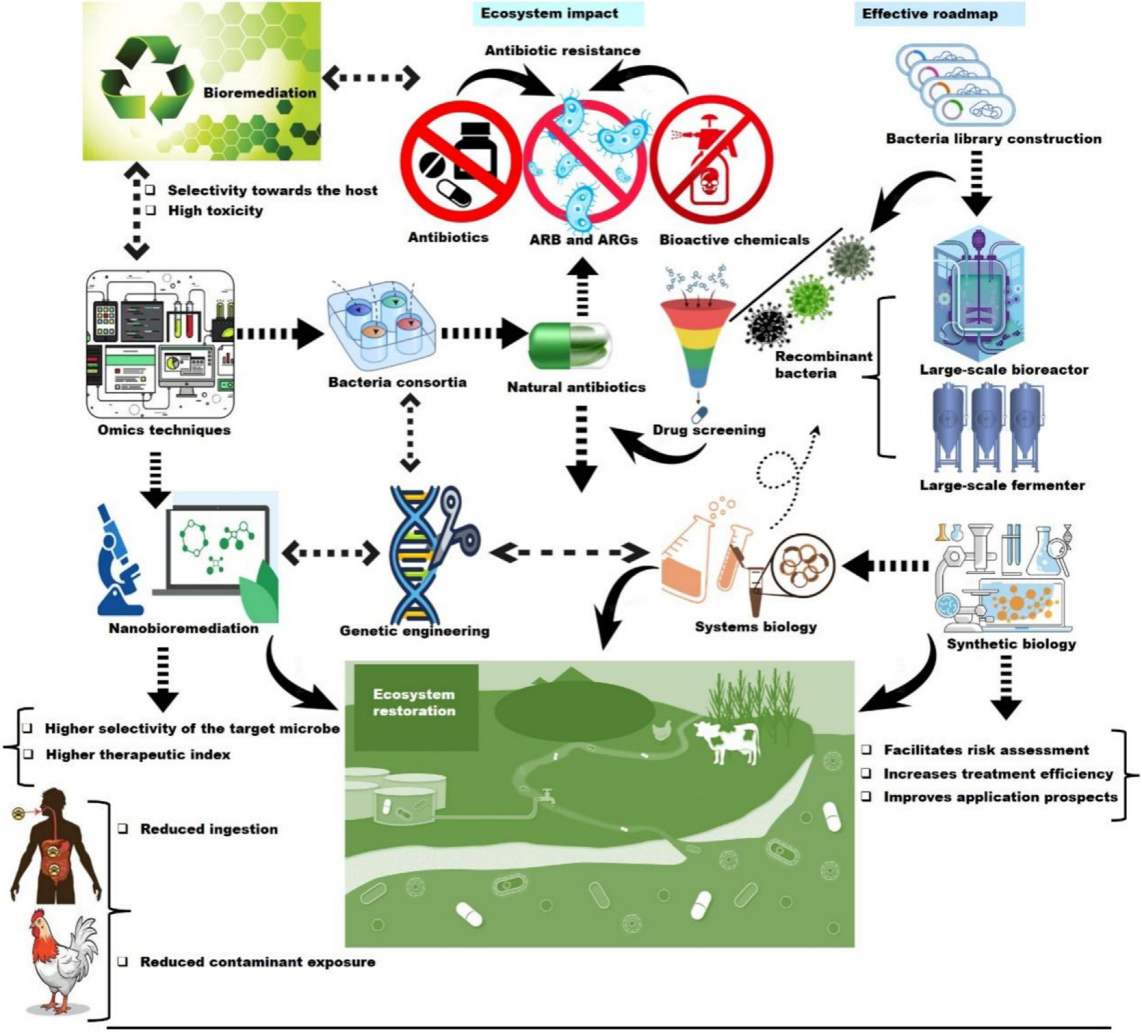
A schematic of a roadmap for abating the interactive impacts and ecological risks of antimicrobials and bioactive chemicals in agroecosystems. The proposed roadmap outlines a systematic approach to mitigate the interactive impact of antimicrobials and bioactive chemicals in agroecosystems by emphasizing source control, efficient monitoring, and adopting advanced bioremediation technologies. By integrating these strategies, a science-based framework could potentially reduce the impact of these residues in agroecosystems for sustainable agricultural practices and ecosystem restoration.

Antimicrobial residues in agriculture increase antimicrobial resistance globally. The interactions between antimicrobial residues, bioactive chemicals, and their consequences elucidate a complex web that sustains ARGs and bacteria. However, the coexistence of antimicrobials and bioactive chemicals in agroecosystems revealed their impacts on resistance dynamics, emphasizing the urgent need for sustainable solutions. Nevertheless, understanding how AMR spreads in agroecosystems requires extensive research, which could begin as a roadmap for abating the interactive impacts of these chemicals in agroecosystems (Fig. 7).

Since the first antibiotic was discovered, they have shown to be a handy tool in the battle against microbial infections in humans and animals. However, the significant improvements in health that have been made possible by antibiotic use are in jeopardy due to the rapid rise in AMR, which persists and is difficult to remove. Although some antibiotics are currently used with other antimicrobials or bioactive chemicals to abate resistance, it is anticipated that in the future, researchers may exhaust relative alternatives due to the inability of most pharmaceutical industries to discover and establish novel antibiotics. Therefore, discovering different aspects of natural antimicrobials, such as their selectivity of the target microbe rather than the host (lower toxicity, higher selectivity) and their therapeutic index, which indicates the ratio of a toxic dose to a therapeutic dose, are pertinent. Since the synthesis of naturally occurring antimicrobials is limited, creating recombinant strains with higher yields through genetic engineering and constructing large-scale bioreactors and fermenters could aid in the large-scale production of natural antibiotics.

Furthermore, the survival, activity, and interactions of native microbial communities with non-native microorganisms are critical to the success of bioremediation. Also, it is necessary to investigate the significant role that these bacteria play in the bioremediation of antimicrobials and bioactive compounds, as well as their catabolic potential with regard to genes, enzymes, and degradation pathways. Since most natural microbiomes are still uncultivated, more research can be done using advanced bioremediation techniques to understand these organisms' fundamental role in ecosystems. More effective outcomes can be achieved with advanced bioremediation techniques that are still largely unexplored. For example, omics technologies and bioinformatics tools have been used recently to unveil the way to research breakthrough in the bioremediation prospects of different microorganisms [152], [188]. Nonetheless, their integration with advanced technologies, such as genome editing tools, nanotechnology, and synthetic and systems biology, could be used to efficiently exploit the ability to remove high levels of emerging contaminants. Typically, constructing genetically engineered bacteria strains or designing microbial consortia with the ability to create, synthesize, and enhance various biological functions will facilitate efficient bioremediation of multiple contaminants and tackle AMR in agroecosystems.

## Declaration of competing interest

The authors declare that they have no known competing financial interests or personal relationships that could have appeared to influence the work reported in this paper.
